# Unique Roles of Gold Nanoparticles in Drug Delivery, Targeting and Imaging Applications

**DOI:** 10.3390/molecules22091445

**Published:** 2017-08-31

**Authors:** Fen-Ying Kong, Jin-Wei Zhang, Rong-Fang Li, Zhong-Xia Wang, Wen-Juan Wang, Wei Wang

**Affiliations:** School of Chemistry and Chemical Engineering, Yancheng Institute of Technology, Yancheng 224051, China; kongfy@ycit.edu.cn (F.-Y.K.); zhangjinwei18@163.com (J.-W.Z.); Lirongfang2324@163.com (R.-F.L.); wangzx198411@163.com (Z.-X.W.); wwj@ycit.edu.cn (W.-J.W.)

**Keywords:** gold nanoparticles, drug delivery, molecular nanoprobes, imaging, disease diagnosis

## Abstract

Nanotechnology has become more and more potentially used in diagnosis or treatment of diseases. Advances in nanotechnology have led to new and improved nanomaterials in biomedical applications. Common nanomaterials applicable in biomedical applications include liposomes, polymeric micelles, graphene, carbon nanotubes, quantum dots, ferroferric oxide nanoparticles, gold nanoparticles (Au NPs), and so on. Among them, Au NPs have been considered as the most interesting nanomaterial because of its unique optical, electronic, sensing and biochemical properties. Au NPs have been potentially applied for medical imaging, drug delivery, and tumor therapy in the early detection, diagnosis, and treatment of diseases. This review focuses on some recent advances in the use of Au NPs as drug carriers for the intracellular delivery of therapeutics and as molecular nanoprobes for the detection and monitoring of target molecules.

## 1. Introduction

Recently, the application of nanotechnology as a new method for the diagnosis, monitoring, and cure of disease has garnered attention in the biomedical field [1]. Nanomaterials, with a size range from 1 to 1000 nm in diameter, exhibit several unique properties that differ greatly from those observed in fine particles or bulk materials. They have potential for many biomedical applications, due to their large specific surface, high surface activity, strong antioxidant property, good biocompatibility, and suitableness for manipulations at the molecular level. At present, common nanomaterials used in biomedical applications include liposomes, polymeric micelles, graphene, carbon nanotubes, quantum dots, magnetic nanoparticles, metallic nanoparticles, and so on. Their use has been shown to significantly enhance therapeutic outcomes.

Among the various nanomaterials described above, the biomedical use of metallic nanoparticles, especially gold nanoparticles (Au NPs), has peaked interests as they offer manifest advantages. Firstly, we can easily synthesize various shapes of Au NPs with sizes ranging from 1 nm to more than 100 nm, such as spherical, rod-like, cage-like, and so on. The optical and electrical properties of Au NPs greatly depend on their shape and size [2]. Secondly, due to the presence of a negative charge on Au NPs, they can be easily functionalized by all kinds of biomolecules, such as drugs, genes, and targeting ligands [3]. Thirdly, Au NPs are biocompatible and nontoxic [4]. Fourthly, Au NPs have distinct surface effect, ultra-small size, macroscopic quantum tunneling effect, and the presence of surface plasmon resonance (SPR) bands [5]. All of these particular properties have caused Au NPs to become the most potential material for various biomedical applications, including biosensing, molecular imaging, drug carriers, and so on. Extensive information on the most important aspects of the preparation and use of Au NPs in biosensing have been published elsewhere [6,7,8]; this review will narrowly focus on the latest research in which Au NPs have been applied for use in molecular imaging and drug carriers for disease therapy.

## 2. Synthesis and Biofunctionalization

### 2.1. Synthesis

Au NPs can be synthesized by a variety of methods. Detailed reviews involving the synthetic procedures for Au NPs have been reported [9]. Here, we will give a brief review. Au NPs synthesized for biological applications are commonly prepared using the colloidal synthesis method. This approach utilizes a metal precursor, a reductant, and a stabilizer, and allows for the facile tuning of the size, shape, and optical properties of the nanostructures. Some of the syntheses for Au NPs are highlighted below.

Spherical gold nanoparticles (Au NSs) are one of the most widely used gold nanostructures in drug-delivery applications. A large number of Au NSs can be easily synthesized with relatively high single dispersion by the reduction of aqueous chloroauric acid with sodium citrate. By varying the stoichiometric ratio between chloroauric acid and sodium citrate, the size of the spherical nanoparticles can be controlled [10]. In this synthesis, citrate not only acts as the reducing agent, but also acts as a stabilizer.

Gold nanorods (Au NRs) are another commonly used gold nanostructure in photothermal and near infra-red (NIR) applications. There are two general colloidal approaches to Au NRs synthesis: Seed-mediated and seedless growth. The seed-mediated growth method often requires a solution of small seeds (3–5 nm). The seeds serve as nucleation sites for Au^+^ anisotropic reduction to form Au NRs. The length and the aspect ratio of the Au NRs can be adjusted by increasing the concentration of AgNO_3_ [11]. The seedless growth method requires an acidic pH of growth solution, and reducing agent is added to simultaneously initiate seed formation and Au NRs growth [12].

### 2.2. Biofunctionalization

Surface functionalization is one of the most favorable properties of Au NPs in the biomedical domain. The surface of Au NPs can be functionalized with various biomolecules, such as DNA, peptides, and antibodies. There are two kinds of interaction. One is noncovalent interactions, the other is covalent interactions. Noncovalent modifications take place through electrostatic interactions, hydrophobic entrapment, and van der Walls forces. The advantage of this interaction is that the biomolecule cannot attached to various chemical modifications that are likely to compromise its primary, active modality. However, the binding is not strong enough to yield stable surfaces capable of suffering the necessary washing steps and incubation conditions, especially in biological studies. So, it is important to consider the impact of the surrounding medium’s ionic strength and pH when using this modification. In comparison, covalent modifications, which make use of immediate chemical attachment, linker molecules, or click chemistry, provide greater stability and reproducibility [9]. Covalent modifications can withstand a very high salt concentration and are extremely stable under thermal conditions. However, covalent modifications are normally more complex, sometimes requiring intensive synthesis work on the ligands. The relative ease of nanoparticle surface modification, through noncovalent and covalent modifications, allows for specific biological targeting, and further can be used in biodiagnostic and biosensing applications.

## 3. Au NPs as Drug Delivery Carriers

Drug delivery is an intriguing field of research that has attracted the attention of researchers. We can define drug delivery as a process for the release of biologically active medicament at a certain speed and at a specific location. At present, it is vital to ameliorate specific drug delivery modes for clinical application. Nanomaterials offer enormous probabilities for multiple, locus-specific drug delivery to the disease locus as their diminutive size can effectively penetrate across obstacles through small capillaries into individual cells. Specifically, Au NPs have revealed great capacity for use as drug delivery platforms. Au NPs can deliver multiple drugs molecules, recombinant proteins, vaccines, or nucleotides into their targets and can control drug release via biological stimuli (internal) or light activation (external).

### 3.1. Au NPs for Drug Delivery

**Figure 1:**
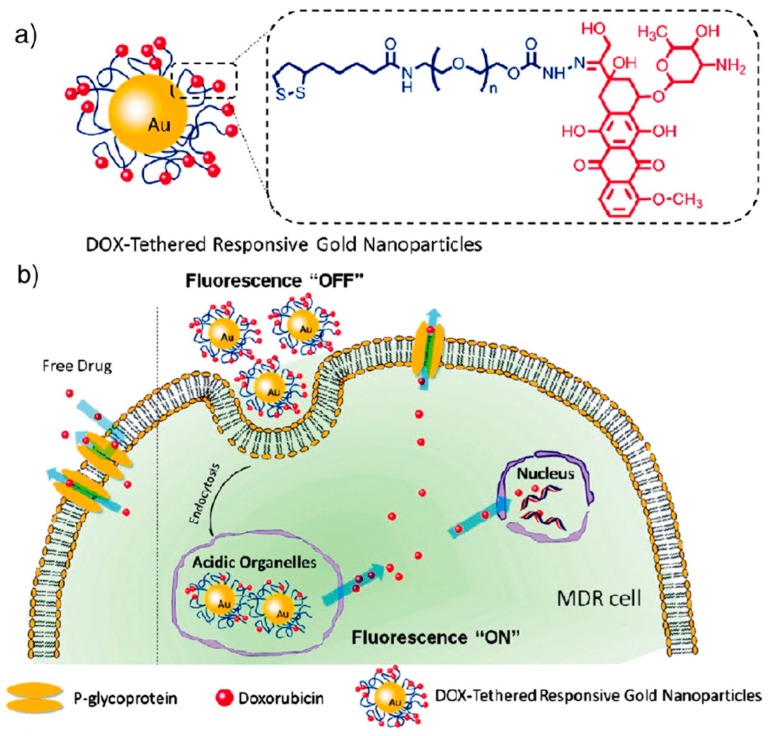
(**a**) Schematic illustration of DOX-tethered responsive gold nanoparticles (Au NPs); (**b**) Schematic illustration of the cooperation between enhanced DOX cellular entry and a responsive intracellular release of DOX into the cells to overcome drug resistance [16]. Reprinted with permission from reference [16]. Copyright (2011) American Chemical Society.

Conjugates of Au NPs with drug molecules play an important role in the therapy of endocellular diseases [13,14]. They could improve drug efficacy. Antibiotics or other drug molecules are able to directly conjugate with Au NPs via ionic or covalent bonding, or by physical absorption. For instance, 13 nm colloidal Au has been combined to methotrexate [15]. Methotrexate is an analog of folic acid which has the ability to restrain the growth and reproduction of cancer cell and has been usually used as an anticancer drug. The carboxylic groups on the methotrexate molecule can combine to the surface of Au NPs after overnight incubation. At the same volume, it has been indicated that the concentration of the methotrexate bound to Au NPs is higher than that of the absence of Au NPs. In another study, doxorubicin (DOX) was attached to 30 nm Au NPs through a pH-sensitive linker. This type of DOX-Au NP attachment allows for the intracellular triggered release of DOX from the Au NPs once inside acidic organelles. This allowed for a rapid increase in intracellular DOX concentration, thereby enhancing therapeutic effects in drug-resistant tumor cells [16] (Figure 1).

The surface of Au NPs can be modified by using poly ethylene glycol (PEG) as a spacer. The amphiphilic characteristics of polymers ensure Au NPs excellent stability in physiological conditions and provide a multiformity of combinations on Au NPs. A great many studies have manifested the excellent properties of polymer-modified Au NPs. Wheate et al. attached the active ingredients of the anticancer drug oxaliplatin to Au NPs for improved drug delivery [17]. Naked Au NPs were modified with a thiolated PEG monolayer capped with a carboxylate group. [Pt(1*R*,2*R*-diaminocyclohexane)(H_2_O)_2_]_2_NO_3_ was loaded to the PEG surface to produce a supramolecular compound. The platinum-tethered nanoparticles were tested for cytotoxicity, drug intake, and localization in the A549 lung epithelial cancer cell line as well as in the HCT116, HCT15, HT29, and RKO colon cancer cell lines. The platinum-tethered nanoparticles revealed as good as, or markedly better, cytotoxicity than single oxaliplatin in all of the cell lines, in addition to a unique ability to penetrate the nucleus in the lung cancer cells. Au NPs combined with courmarin-PEG-thiol were yielded and found to be quickly internalized into cells by the action of non-specific endocytosis [18]. Bhattacharya et al. found that Au NPs interact with PEG-amines folic and acid by noncovalent bonds were easily targeted to the folate receptors of cancer cells. The authors noted that polymer-drug conjugates of folic acid with Au NPs could have a great effect on folate receptor-targeted drug delivery or targeted therapy in the future [19]. Gu et al. used 3-mercaptopropionic acid (MPA) to modify spherical Au NPs. NH_2_-PEGNH_2_ was then combined to the MPA layer via amidation between the carboxylic group on the Au NPs and the amine end-groups on the PEG. This conjugation leads to a fine stability in an electrolyte environment and a high efficiency of intracellular transport, which are significantly useful for delivery targeted to the nucleus [20].

### 3.2. Au NPs for Gene Delivery

Gene therapy presents an ideal strategy for the treatment of genetic as well as acquired diseases [21]. Recent studies have suggested that, as they are capable of delivering all kinds of oligonucleotides such as plasmids, double stranded DNA (dsDNA), single stranded DNA (ssDNA), and single stranded RNA (ssRNA) could have excellent therapeutic effects. Au NPs provide attractive candidates for DNA and RNA delivery. Au NPs protect nucleic acid and prevent them from being degraded by nuclease. They can help nucleic acid transfect cells and play a role in targeting. At present, Au NPs with a variety of morphologies, such as nanospheres and nanorods, have been used for this purpose.

Oligonucleotide (and small interfering RNA)-modified Au NP conjugates have unique properties that make them potential intracellular gene regulatory agents. Au NPs firmly functionalized with covalent bonding of oligonucleotides are able to activate immune-related genes and pathways in peripheral blood mononuclear cells of the human body, but not a permanent, lineage-restricted cell line. These findings have vital value for the application of oligonucleotide-modified Au NPs conjugates in translational research and in the development of therapy and gene delivery technology [22].

As early as 2001, there was a survey of Au NPs functionalized with cationic quaternary ammonium groups and electrostatically incorporated into the plasmid DNA. The results demonstrated that the composite particle could protect the DNA from enzymatic degradation and regulate DNA transcription of T7 RNA polymerase [23,24]. Mirkin et al. coated citrate-stabilized spherical Au NPs with a dense layer of ssDNA molecules functionalized with either single or multiple thiol groups and used them in gene silencing applications [25]. This conjugate was internalized efficiently by cells, and the structures were able to resist degradation by proteases and a high level of endogenous glutathione levels. The integration of complementary DNA with the grafted ssDNA was enhanced, which result in the increased efficacy of the drugs in gene silencing applications.

**Figure 2:**
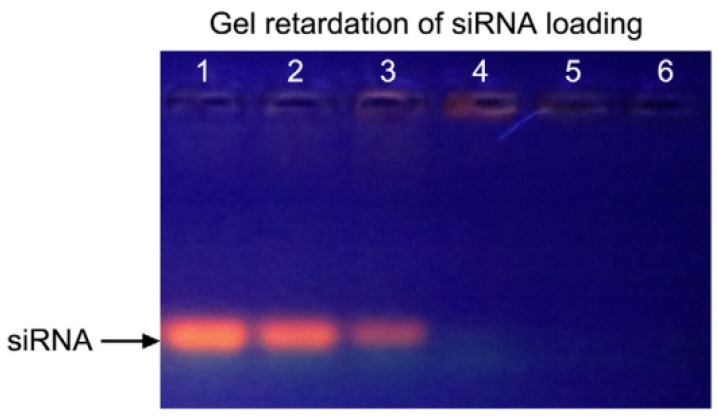
The quantitative analysis of gold nanorods (Au NRs) for the delivery of siRNA. Agarose gel electrophoresis was used to detect the bonding between siRNAs and Au NR-PSS-PAH [27]. Reproduced with permission from reference [27]. Copyright (2015), with permission from Elsevier.

Owing to the size- and shape-dependent optoelectronic properties of Au NRs, they also have the capability to deliver siRNA to target cells or tissues. Prasad’s group recently conjugated Au NRs and cetyltrimethylammonium bromide (CTAB) to siRNA (against DARPP-32 gene in dopaminergic neuronal (DAN) cells) and studied the intake of conjugates interior the DAN cells [26]. By means of dark-field imaging and confocal microscopy, it was revealed that the Au NR-siRNA conjugates are able to efficiently deliver the siRNA to DAN cells, and cell viability was 98%. The results of study have also verified that Au NPs can be applied as novel carriers to deliver genes into neuron cells. Zhao et al. developed Au NR-based nano-carriers with poly-sodium 4-styrenesulfonate (PSS) and poly-allylamine hydrochloride (PAH), which are able to deliver small interfering RNA (siRNA) against LSD1 to induce the differentiation of human mesenchymal stem cells (MSCs) [27] (Figure 2). The results of their study manifested the successful internalization of Au NR-PSS-PAH-siRNA (Au NRssiRNA) nano-carriers, which restrains the manifestation of LSD1 and prompts the differentiation of human MSCs into a hepatocyte lineage in vitro with hepatocyte growth factor (HGF). Their research achievements may contribute to develop more useful nano-platforms to deliver siRNA for tissue regeneration therapy.

### 3.3. Au NPs for Protein Delivery

Au NPs can also be used as nano-carriers for protein delivery. The interfacial interaction between protein and Au NPs has profound insinuations for the applications of Au NPs in biology and biomedicine. Organothiol is an influential molecular probe used to study the structure, morphology, and stability of proteins on Au NPs. With its high relative abundance in biofluids containing serum plasma, the probability of the inclusion of organothiol into protein-coated Au NPs and its potential impact on the functionality and toxicity of the protein-covered Au NP should not be ignored in biological and biomedical fields [28]. In previous studies, Au NPs have been functionalized by chitosan for delivering insulin [29]. Chitosan is a kind of nontoxic biopolymer that can stabilize Au NPs. Chitosan-coated particles strongly adsorb insulin on their surface, and are effective for the transmucosal delivery of insulin. Rotello et al. reported that Au NPs functionalized by cationic tetraalkyl ammonium recognize the surface of an anionic protein through complementary electrostatic interaction and restrain its activity [30]. The activity was improved on account of the release of free protein by treating the protein-particle complex with SH, displaying Au NPs as potential protein transporters. Krol et al. used Au NPs to conjugate either with human serum albumin (alb-Au NP) or apolipoprotein E (apoE-Au NP) prior to intravenous injection. The results showed that protein conjugation massively reduced liver retention when compared to citrate-stabilized Au NPs. Their study clearly suggests that the stable conjugation of Au NP with albumin and apoE prior to intravenous administration increases the specificity and efficiency of NPs in diseased target organs, thus suggesting a potential role in nanomedicine and nanopharmacology [31].

### 3.4. Au NPs for Vaccine Delivery

Prophylactic vaccination is one of the most effective interventions in medicine and is responsible for substantial decreases in morbidity and mortality by many pathogens worldwide. Traditional vaccines are remarkably effective, but there are limitations to their production and distribution. Recently, Au NPs have been extensively used as vaccine platforms which have potential advantages over traditional vaccine platforms, due to their varying sizes, shapes and tunable surface properties. Nebaikina et al. proposed the use of Au NPs in designing vaccines against tick-borne encephalitis [32]. An appropriate design made Au NPs multivalent, which enhanced their interactions with the target receptors. Chen’s group used three surface-engineered Au NRs as HIV-1 Env plasmid DNA vaccine adjuvants for human immunodeficiency virus (HIV) treatment [33]. Shiang et al. developed aptamer (Apt)-conjugated Au NPs (Apt-Au NPs, 13 nm in diameter) as highly effective inhibitors for the HIV reverse transcriptase [34]. Chen et al. reviewed the use of Au NPs in HIV/acquired immune deficiency syndrome (AIDS) vaccine development [35]. Up to now, many excellent reviews have reported on the topic [36,37,38,39,40].

### 3.5. The Release of a Drug from Au NPs via External Stimulus

The release of a payload in a spatiotemporal fashion has a substantial impact on increasing therapeutic efficacy. Because of their unique physical, chemical, and optical properties, Au NPs can be used in new methods to control the delivery and release of drugs [41], using external stimuli (operated with the support of stimuli-generating processes, such as the application of light) or internal stimuli (operated within a biologically controlled manner, such as pH or glutathione) to release the drug from the Au NPs [42,43].

Upon external stimuli, light is mostly used to release drug molecules from Au NPs. For example, Chompoosor et al. demonstrated a novel monolayer of Au NPs that was used to study the controlled release of a model drug using UV light [44]. Hydrophobic molecules were noncovalently entrapped in the compartments of its monolayers. Once irradiated with UV light, the dinitrobenzyl linker was cleaved, leading to the release of the entrapped agent. UV light provides a higher release of drugs compared to a system exposed to no UV radiation. The release of dye can be controlled by controlling UV irradiation. Li and his colleagues applied a combination of chemotherapy and photothermal ablation for the treatment of metastatic breast cancer using DNA-coated Au NRs (GNR) with co-delivered DOX (GNR@DOX) [45]. Upon laser irradiation, the local temperature of GNR@DOX was increased substantially, and efficiently released the laden DOX. Caruso et al. used microencapsulation technology to encapsulate macromolecules just as fluorescent labeling dextran [46]. Au NP-doped capsule-shells, which are able to resist near infra-red (NIR) light. Due to the rupture of the shell, fluorescein isothiocyanate-dextran was liberated upon laser (1064 nm) treatment. Zhang and his fellows found a core/shell nanoparticle that consists of an Au nanoshell and 10-hydroxycamptothecin (HCPT-NPs) for the treatment of breast cancer [47]. At an irradiation power density of 1 W cm^−2^ for 10 min, the core-shell nanoparticle resulted in full tumor remission in a 4 T1 breast syngeneic mouse model and no significant weight loss of mouse and tumor recurrence. Choi and co-workers developed a PEGylated multifunctional hollow gold nanoparticle (HGNP) delivering DOX (DOX-HGNP) for the therapy of A549 lung cancer [48]. Upon NIR irradiation, The NPs can efficiently release DOX. Furthermore, HGNP was capable of sensitizing A549 cells to 6 MV X-ray radiation, as evidenced by the radiation-induced DNA double-strand breaks in A549 cells. The effective combination of DOX, high temperature, and radiation showed the highest level killing of the cancer cells in vitro. In another study, silicon phthalocyanine 4 (Pc 4), a drug for the treatment of rabies, was bound to PEGylated Au NP conjugates. After the PEGylated Au NP-Pc 4 conjugate reaches the tumor site, the Pc 4 molecules are liberated from the surface of the nanoparticle and start phototherapy via irradiation with light of about 670 nm [49].

Another external stimulus that can be used for the delivery of genes from an Au NPs is electroporation. Kawano et al. used electrical pulses to stimulate Au NPs to explore gene transfer in vivo [50]. In their research, Au NPs were integrated with plasmid DNA and modified with mPEG-SH5000. They then injected the conjugates into the anesthetized mice. In order to ensure that the conjugates were adequately diffused in the mice, electrical pulses were then used to the left lobe of the liver after a certain period of time. The result showed that there was gene expression in the organs of major mice. In contrast, the mouse injected with DNA without any modification lead to a tenfold reduction in detection. The degradation time of DNA in blood was as short as 5 min, and it was a clear reason for the low transfection efficiency in the latter case. This research showed a novel stimulating way to perfect gene delivery applying Au NPs.

## 4. Au NPs as Molecular Nanoprobes

### 4.1. Au NP-Based Molecular Diagnostics

**Figure 3:**
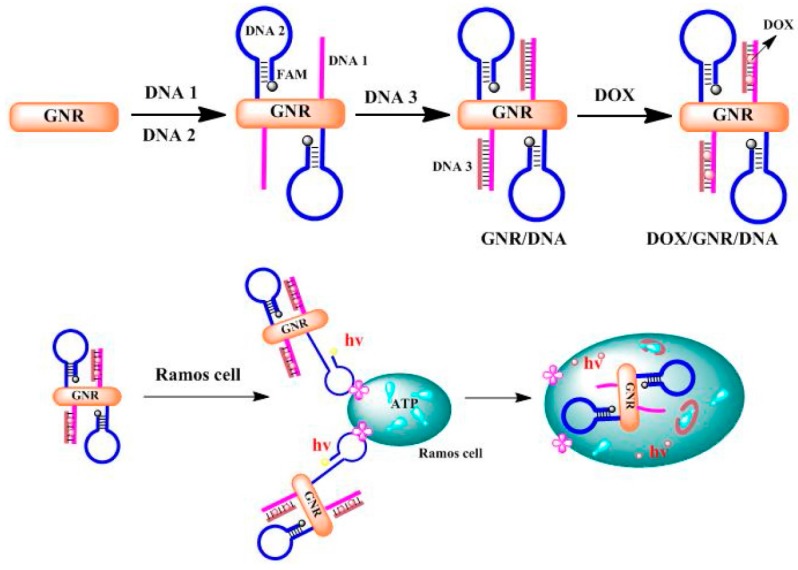
A targeted, delivery-quantification DNA assembly platform. This platform consists of Au NRs, DOX, and DNA sequences, which are composed of aptamer DNA strands and their complementary DNA strands [52]. Reproduced with permission from reference [52]. Copyright (2016), with permission from Elsevier.

The appearance of Au NPs has greatly improved the sensitivity, specificity, multiplexing, and turnaround times of molecular diagnostics. The widespread use of Au NPs as sensing interfaces and as labels for signal amplification in bio-recognizing events have been reviewed elsewhere. Here, we will briefly introduce Au NPs as molecular nanoprobes for diagnostic applications. For example, based on the conjugation of a fluorescent DNAzyme onto Au NPs, Lu et al. developed the first DNAzyme-based metal sensors for intracellular metal ion detection [51]. The probe was composed of a 13 nm Au NP core coated with a shell which was comprised of a uranyl-specific 39E DNAzyme. The authors demonstrated that these DNAzyme-Au NP probes can easily access cells and act as a metal ion sensor within a cellulate condition. Continued development of these DNAzyme-Au NP probes will allow for a better understanding of the localization and distribution of metal ions in biological systems. Guo et al. developed aptamer switch probes with dual functions based on Au NRs for targeted cancer treatment [52] (Figure 3). In their work, Ramos cell aptamer and ATP aptamer were immobilized on the surface of Au NRs. When the above two aptamers were covered on the Au NRs, they presented a high specific recognition ability for tumor sites. At the same time, various GC-pair sequences into ATP aptamer dsDNA were designed to increase the carrying ability of the drug DOX. The detection of intracellular ATP and the delivery of drug molecules into target cells was achieved by combining the roles of two functional aptamers. Au NP nanocomposites have also been employed as sensing interfaces and as label ultrasensitively detected the carcinoembryonic antigen [53,54].

### 4.2. Au NP-Based Molecular Imaging

In the past 10 years, molecular imaging technology has developed rapidly, which are able to provide physiological and pathological information with high sensitivity and specificity for disease diagnosis. A variety of imaging types such as optical imaging, computed tomography (CT) imaging, ultrasound imaging, and magnetic resonance (MR) imaging have been developed for disease diagnosis. Herein, we highlight surface-enhanced Raman scattering (SERS) and CT imaging.

Compared with other imaging techniques, SERS has the advantages of high sensitivity, high accuracy, no label, no damage, and no invasion for molecular identification. In the SERS method, Au NPs usually have been used as intracellular probes to monitor intracellular drug release as well as to probe molecules by targeting them to cellular compartments such as mitochondria, endosomes, and the cell nucleus [55]. Fabris discovered a novel type of tags with good biocompatibility upon dimeric assemblies of spherical Au NPs for specific tumor targeting and SERS-based detection [56]. Compared with the monomer, the innovation of a narrow spacing between the nanoparticles on dimerization led to more pronounced signals, longer activity, and lower cytotoxicity. It was proven that cell-internalized Au NP dimers possess high specificity for cellular recognition and prominent stability. Hence, SERS-based cell detection is faster and more sensitive than traditional fluorescence imaging. Hu’s group developed a new nuclear targeting nanoprobe to be based upon peptide functionalized Au NPs employed with SERS in living cells [57]. In their report, the SV-40 large T nuclear localization signal (NLS) peptide integrated with Au NPs triumphantly accessed the cell nucleus after incubation with Hela cells and transmitted the spatially localized chemical information of the nucleus, as well as the signature of chemicals that subsequently intruded. This novel targeted nanoprobe is a good method with nontoxic, biocompatible advantages and has the potential to be used in the research of drug delivery and cancer therapy.

**Figure 4:**
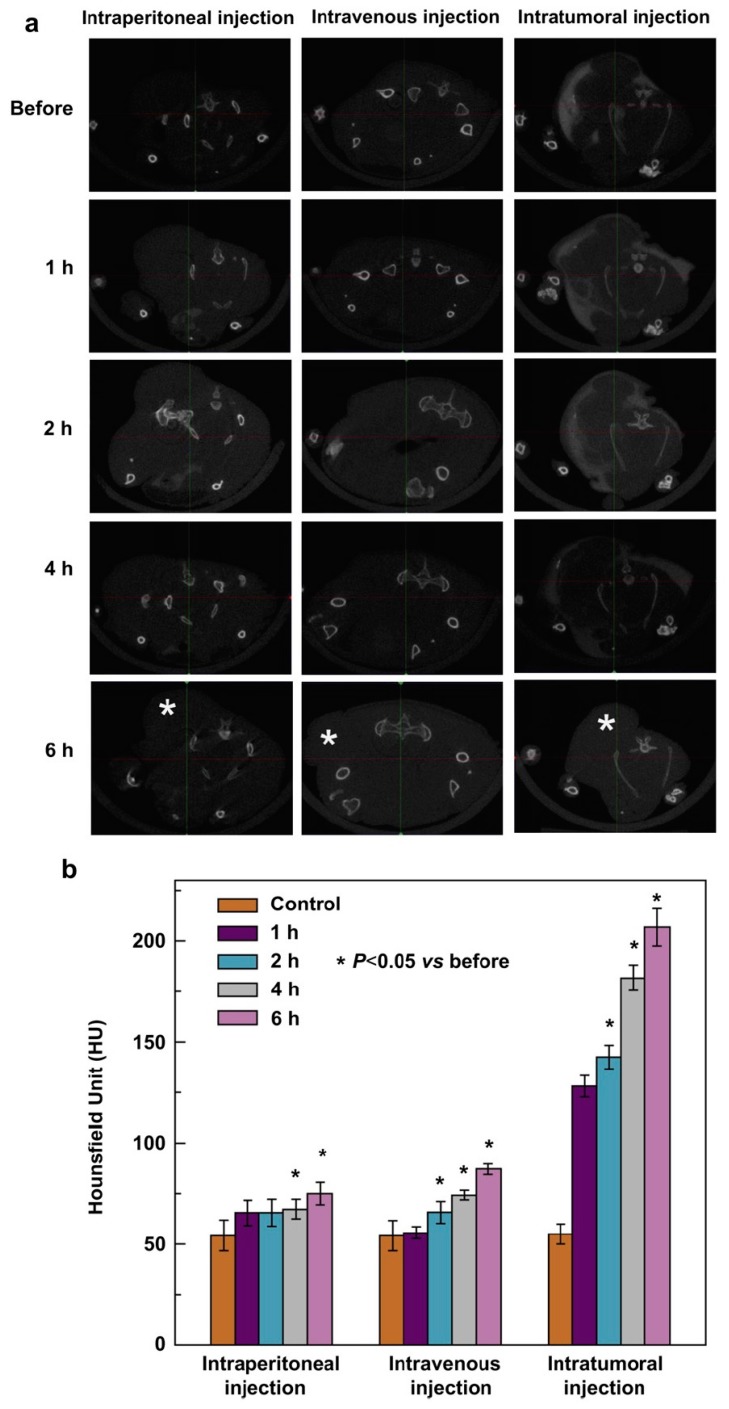
Representative axial micro- computed tomography (CT) images (**a**) and CT values (**b**) of the xenograft SPC-A1 tumor in nude mice before and after injected with {(Au^0^)_50_-G5.NHAc-FA_5_} DENPs by different injection routes for 1, 2, 4, and 6 h. The star “*” in (a) indicates the location of the tumor [63]. Reproduced with permission from reference [63]. Copyright (2013), with permission from Elsevier.

Widely used in clinical applications, CT has the unparalleled advantages of high spatial resolution and unlimited penetration depth [58]. Au NPs have unique physical, chemical, and biological properties, which make them an ideal candidate for CT contrast agents [59]. Kim et al. conjugated the A10 aptamer, targeted against prostate specific membrane antigen (PSMA), to Au NPs [60]. They showed that A10-targeted nanoparticles can bind to PSMA-expressing prostate cancer cells with high sensitivity and specificity. Furthermore, they found that A10-Au NP conjugates can be used as a molecular probe for CT imaging to detect PSMA-expressing cancer cells. Zhang’s group utilized G5.NH_2_ dendrimers as templates to synthesize dendrimer-entrapped gold nanoparticles (Au DENPs) [61]. The acetylation of terminal amine groups form Au DENPs with a neutral surface. Acetylated Au DENPs could be used as CT imaging agents for a model cancer cell, SPC-A1, in vitro and for a xenograft tumor model of nude mice in vivo. Recently, they presented the synthesis and characterization of Au DENPs modified by PEG, which have excellent biocompatibility and potential applications for CT imaging [62]. The PEGylated Au DENPs enabled not only CT blood pool imaging of mice and rats after intravenous injection of the particles, but also effective CT imaging of a xenograft tumor model in nude mice. More recently, they reported a new role of Au DENPs (Au DENPs-FA) modified by folic acid as nanoprobes for in vitro and in vivo targeted CT imaging of human lung adenocarcinoma [63] (Figure 4). MicroCT images display that SPC-A1 cells are observable under X-ray after incubation with the Au DENPs-FA in vitro. Moreover, the xenograft tumor model was observable through imaging after intratumoral, intravenous, and intraperitoneal administration of the granules.

Dual mode or multimode imaging is essential for accurate or self-confirmation disease imaging. Shi et al. reported the synthesis, characterization, and applications of gadolium-loaded Au DENPs (Gd-Au DENPs) for dual mode CT/MR imaging [64]. One NP system containing two radiodense imaging elements of Au NPs and Gd (III), Gd-Au DENPs, showed both r1 relaxivity for MR imaging mode and X-ray attenuation for CT imaging mode, which contribute to enable the CT/MR dual mode imaging of the heart, kidney, liver, and bladder of mouse or rat within a time frame of 45 min. Zhang et al. reported an excellent nanoprobe (M-NPAPF-Au) co-loading Au NPs and an aggregation-induced emission (AIE) red dye into DSPE-PEG2000 micelles for dual-modal fluorescence/CT imaging [65]. In vitro and in vivo results showed that the nanoprobe has favorable practicability with good biocompatibility, superior tumor-targeting ability, long blood circulation half-life, and prominent fluorescence and CT imaging functions.

## 5. Conclusions

As highlighted in this review, there has been significant interest and research conducted on Au NPs for drug delivery, imaging, and biodiagnostic applications. Their biologically relevant size, low inherent toxicity, high surface area, and ability to easily functionalize with biomolecules, as well as their enhanced optical properties provides them with unique attributes that help to improve therapeutic delivery, imaging, and noninvasive disease diagnostics. In future work, efforts need to be devoted to construct multifunctional Au NPs by the conjugation of various targeting molecules. The Au NP formulations need to be fine-tuned and tailored to obtain optimized therapeutic combinations. Modeling of Au NPs with desired properties and subsequently developing a procedure to synthesize the theoretically predicted nanostructure is also warranted. Finally, Au nanostructures’ long-term cytotoxicity, genotoxicity, and in vivo and in vitro targeting efficiency also should be evaluated.
